# Simulations of the response of supported 2D materials to ion irradiation with explicit account for the atomic structure of the substrate

**DOI:** 10.1039/d5na00468c

**Published:** 2025-08-13

**Authors:** Mitisha Jain, Silvan Kretschmer, Arkady V. Krasheninnikov

**Affiliations:** a Institute of Ion Beam Physics and Materials Research, Helmholtz-Zentrum DresdenRossendorf 01328 Dresden Germany m.jain@hzdr.de a.krasheninnikov@hzdr.de; b CAMD, Computational Atomic-scale Materials Design, Department of Physics, Technical University of Denmark 2800 Kongens Lyngby Denmark

## Abstract

Ion irradiation has routinely been used to create defects or even pattern two-dimensional (2D) materials. For efficient defect engineering, that is, choosing the proper ion fluence to achieve the desired concentration of defects, it is of paramount importance to know the probability of creating defects as a function of ion energy. Atomistic simulations of ion impacts on 2D targets can provide such information, especially for free-standing systems, but in the case of supported 2D materials, the substrate can strongly affect defect production. Here, we employ analytical potential molecular dynamics simulations to calculate the average number of defects produced by light (He) and heavy (Ar) ions in 2D MoS_2_ and graphene, two archetypal 2D materials, both free-standing and supported, in a wide range of ion energies. We take explicit account of the atomic structure of the SiO_2_ and Au substrates and use several approaches to choose impact points in the supercell to increase the accuracy of the calculations. We show that depending on ion type and energy, the substrate can increase or decrease defect production, and the concentration of irradiation-induced defects and sputtering yield can be quite different for different substrate types. Our simulations provide microscopic insights into different channels of defect production in free-standing and supported 2D systems, and give quantitative results on sputtering yield and defect concentration, which can directly be compared to experimental data.

## Abstract 2

Response of 2D materials to ion irradiation depends not only on ion type and its energy, but also the substrate.

## Graphical abstract

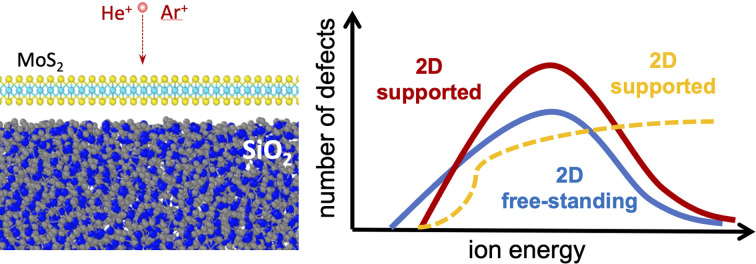

## 1 Introduction

Ion irradiation of two-dimensional (2D) materials has proven to be a powerful method for modifying their properties by creating defects in a controllable manner, adding impurities and even cutting and patterning 2D samples, see ref. 1–5 for an overview. In particular, impurities can be introduced into 2D materials directly by low-energy ion implantation^6–12^ or by filling the vacancies created by energetic particles^13,14^ after irradiation with foreign atoms, which is possible due to the geometry of the 2D system.

As the helium ion microscope (HIM) makes it possible to focus the ion beam into a sub-nm area,^15^ specific attention has been paid to He ion irradiation. Using a HIM, the electronic properties of few-layer MoS_2_ on a SiO_2_ substrate^16,17^ were tuned. Free-standing nanoribbons of MoS_2_ were fabricated,^16^ along with memristors.^18^ The opto-electronic properties of devices made from other transition metal dichalcogenides (TMDs), such as WSe_2_, were tailored by selectively creating defects using focused He ion beams.^19^ Defect-based single-photon emitters have been manufactured in TMDs^20,21^ and h-BN^22^ using HIM.

**Fig. 1:**
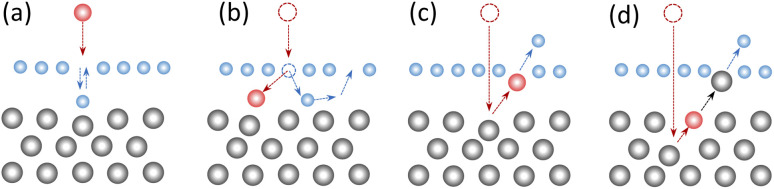
Schematic illustration of the effects of the substrate on defect production in a supported 2D material under ion irradiation. The substrate atoms are colored in grey, the 2D target atom in blue, and the impinging ion is represented as the red circle (filled or empty). The recoil atom sputtered from the 2D material can be reflected by the substrate and immediately be incorporated into the atomic network (a) or diffuse between the 2D material and the substrate and fill a pre-existing vacancy (b). These processes reduce the amount of damage in the irradiated 2D material. The ion can also be backscattered by the substrate (c) or sputter a substrate atom, which in turn displaces an atom from the 2D target. Processes (c) and (d) increase the number of defects in the 2D material.

In most ion bombardment experiments, the irradiated 2D materials were on a substrate, although free-standing (*e.g.*, deposited on a TEM grid) 2D materials have also been studied. It was realized long ago^23–25^ that the response of supported 2D materials to irradiation can be strongly affected by the substrate, as confirmed by numerous experiments.^11,26–29^ It is intuitively clear that depending on ion energy and mass, the substrate may cause a drop in defect production by stopping the atoms displaced from the 2D target, or conversely, increase the number of defects due to backscattered ions and atoms sputtered from the substrate, as schematically illustrated in Fig. 1.

For example, Maguire *et al.*,^30^ using Raman spectroscopy, studied damage in suspended and supported graphene under He and Ne ion irradiation (at 30 keV energy). From the measured spectra, they determined significantly higher defect yields in the supported graphene. Thiruraman *et al.*^28^ investigated the influence of Ga ion irradiation on MoS_2_ and WS_2_ on Si/SiO_2_ (also at 30 keV energy). From their experiments, they found decreased defect density in the supported case as compared to suspended 2D material.

The substrate can also have a strong influence on the annealing of defects due to diffusion of atoms between the irradiated 2D material and substrate. A smaller number of defects in the bottom layer was found in isotopically-labeled bilayer graphene,^31^ which was interpreted as a result of enhanced annealing of vacancies by mobile interstitials between the graphene and substrate.

Moreover, even when very little or no damage is caused by the impinging ions in the irradiated 2D system, the substrate can still influence its properties. For example, mechanical strain in proton-irradiated WS_2_ monolayers was introduced as high-energy protons penetrated the flake and formed bubbles in the substrate. The interplay between gas agglomeration, van der Waals forces binding the monolayer to the substrate planes, and the material′s elastic properties led to the formation of atomically thin, spherically shaped domes and the induced strain resulted in a direct-to-indirect band-gap transition.^32,33^

All of these require a microscopic understanding of the defect formation in supported 2D materials. While simulations^34,35^ of the response of free-standing 2D materials to ion irradiation are relatively easy, the estimates of the amount of damage created in the supported 2D materials by energetic ions and assessments of dopant introduction probabilities are much more complicated.

Zhao *et al.*^23^ studied the role of the SiO_2_ substrate in defect production in supported graphene under ion bombardment with heavy ions (Ar and Si) using MD simulations. Comparison of the damage probability in suspended and supported graphene indicated that the presence of the SiO_2_ substrate lowers the damage probability of supported graphene under low-energy Ar and Si ion irradiation, but enhances defect production at high energies. Wu *et al.*^36^ investigated defect production in stacked 2D MoS_2_ and graphene layers using MD simulations. Their findings revealed that placing graphene beneath the MoS_2_ layer effectively reduces defects in the top MoS_2_ layer compared to free-standing MoS_2_ upon Ar irradiation at energies up to 800 eV. However, introducing an SiO_2_ substrate beneath the heterostructure (MoS_2_/graphene) increases defect production, as the substrate diminishes the stabilizing effects of graphene.

The role of the substrate in damage production in SiO_2_-supported MoS_2_ and graphene monolayers under He, Ne, and Ar irradiation was addressed in detail by Kretschmer *et al.*^24^ The analysis was carried out using a combination of molecular dynamics (MD) and Monte Carlo simulations. Specific attention was paid to He ion irradiation at the typical ion energies (10–30 keV) used in HIM. For such ions the study predicted a higher yield for monolayers in the presence of a substrate. A limitation of this work was the lack of an explicit account of the atomic structure of the substrate in the irradiated heterogeneous system. We stress that atomistic simulations of impacts of He ions with such energies are computationally challenging, as they require large simulation cells (thick substrates) due to the small cross section for displacing the atoms in the target and substrate, so that the ions can go deep into the substrate and then be back-scattered, damaging the supported 2D material.

In this work, using analytical potential MD, we investigate the role of two widely used substrates, Au and SiO_2_, for defect production in monolayer MoS_2_ and graphene under He ion irradiation. The atomic structure of the substrate is explicitly accounted for. We demonstrate that for He ions with energies exceeding 10 keV, a mesh (grid) with a high density of impact points should be used, as the target atom displacement cross section is very small. We also employ a ‘special’ mesh with the points predominantly sampled in the vicinity of the target atoms. For the sake of comparison, we also investigate the behavior of the systems under Ar ion irradiation. We provide quantitative results on sputtering yield and defect concentrations as functions of ion energies, which can directly be compared to experimental data.

## 2 Computational details

We carried out classical MD simulations of He and Ar ion irradiation of a free-standing MoS_2_ monolayer and that on SiO_2_ and Au substrates over a wide range of ion energies (from 20 eV to 40 keV). To describe the interactions between atoms, several analytical potentials were employed, as implemented in the LAMMPS code.^37^ For Mo–S, S–S and Mo–Mo bonds in MoS_2_, the adaptive inter-atomic reactive bond order (REBO)^38–40^ and Stillinger–Weber (SW)^41^ potentials were used. The interactions between atoms in the SiO_2_ substrate were defined by the Tersoff potential.^42,43^ For simulating the Au substrate, the embedded-atom-method (EAM) potential^44,45^ was employed. All ion–target atom interactions, as well as repulsion of the atoms at small separations were described by the screened-Coulomb repulsion ZBL^46^ potential. As we were primarily interested in the formation of vacancies in MoS_2_ upon impacts of energetic ions and recoil atoms, the Mo/S–Si/O or Mo/S–Au interactions were also defined by the ZBL potential.

**Fig. 2:**
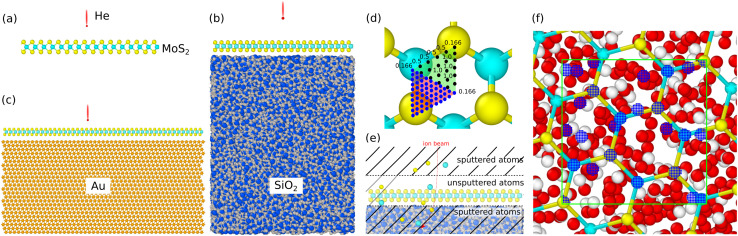
Simulation cells used for suspended MoS_2_ sheets (a), and supported MoS_2_/SiO_2_ (b) and MoS_2_/Au (c) systems. Mo, S, Si, O, and Au atoms are shown in cyan, yellow, blue, grey and orange colors, respectively. The relative atom sizes are not scaled to the true relative vdW radii for better visualization. (d) The illustration of uniform grid of impact points with assigned weights. The black and blue dots represent impact points from sparse and dense grids, respectively. (e) The schematic illustrates the criteria distinguishing sputtered atoms from the displaced ones. (f) Atom-centered sampling (special mesh). Note the very dense grids of impact points (blue dots) on top of the S and Mo atoms, as well as substrate atoms being close to the surface.

The simulation cell dimensions of a suspended MoS_2_ sheet consisting of 810 atoms were 47 × 49 × 50 Å^3^. For the SiO_2_ substrate, a tetragonal unit cell with space group *I*42̄d was taken from the Materials Project.^47^ The unit cell was optimized using the Tersoff potential. The optimized unit cell parameters are *a* = *b* = 5.02 Å and *c* = 7.56 Å. Then the optimized cell was repeated in the *x*, *y* and *z* directions to create a large supercell. The supercell was first heated in the NVT ensemble to 4000 K and then cooled down to 300 K to obtain an amorphous SiO_2_ as described in ref. 48. In the case of monolayer MoS_2_ on SiO_2_ and Au substrates, the cell dimensions were 84 × 59 × 138 and 103 × 119 × 121 Å^3^, respectively. The side views of the simulations cells are shown in Fig. 2(a–c). The substrate thicknesses were around 70 Å (SiO_2_) and 55 Å (Au).

The simulations were performed using the NVE (micro-canonical) ensemble and using adaptive time steps, with the maximum time step being 0.1 fs. The total duration for each simulation varied from about 20 ps to 100 ps depending on the system size and complexity. The simulations were stopped when the energy introduced by the energetic particle was distributed over the whole system. No thermostat region was used for simulations at 0 K, as our test simulations indicated that accounting for energy dissipation at the border has no effect on the production of defects in MoS_2_. Periodic boundary conditions were applied in *x* and *y*-directions with open boundary condition in the *z*-direction.

For the simulations which were performed at elevated temperatures (5 K and 300 K), at first, the NVT ensemble was simulated using the Nose–Hoover thermostat. In the next step, 5 configurations were chosen randomly from the outputs of the previous step; then the ion impact simulations were performed for each configuration. At the end, for each impact point, the results were averaged over these five simulations.

A similar methodology was used for supported graphene upon He and Ar ion irradiation. The interactions in graphene and the SiO_2_ substrate were described by the Tersoff/ZBL potential. In the graphene/Au system, C–C interactions in graphene were defined by the REBO potential^49^ and C–Au interaction was defined by the ZBL potential. The simulation cell size used for suspended graphene irradiation was 129 × 124 × 100 Å^3^. In graphene/SiO_2_ and graphene/Au systems, simulation cells of sizes 168 × 119 × 137 Å^3^ and 112 × 134 × 117 Å^3^ were used, respectively. The substrate depths used were 70 Å (SiO_2_) and 50 Å (Au).

Several uniform grids of impact points with a total number of points *N* were used, as illustrated in Fig. 2(d). The impact points were assigned weights as follows: 1/6 for points at the corners of the triangular region, 1/2 for the points on the edges and 1 for points lying everywhere. The total number of impact points per irreducible area varied from *N* = 105 to *N* = 741.

In addition, for He irradiation of MoS_2_ on SiO_2_ we used a special mesh with impact points localized within a certain radius from the target and substrate surface atoms, Fig. 2(f), and the outcomes of the simulations were re-scaled as a ratio of the areas near the atoms to the total area. We used interaction cross sections predicted from the binary-collision approximation to estimate an effective interaction radius around each atom both in the 2D layer and the substrate (minimum transferred energy *T* = 40 eV). We then uniformly selected a number of impact points in the corresponding circle (typical radius 0.3 Å). The impact point selection was done for all atoms in a rectangular region (area *ca.* 10 × 10 Å^2^) and within 10 Å from the substrate surface. The selection was carried out for the substrate-supported system, but the exact same impact points were also used for the free-standing system (typically *N* = 570 impact points in total), to foster direct comparability. Fig. 2(f) displays the atom-centered sampling. The reason for using such a mesh will be explained later.

## 3 Results and discussion

### 3.1 Effect of temperature on sputtering yield from a free-standing MoS_2_ monolayer

We first address the effects of the number of points in the mesh (grid) and temperature on defect production and sputtering yield from a free-standing MoS_2_ monolayer. The REBO potential was used.

**Fig. 3:**
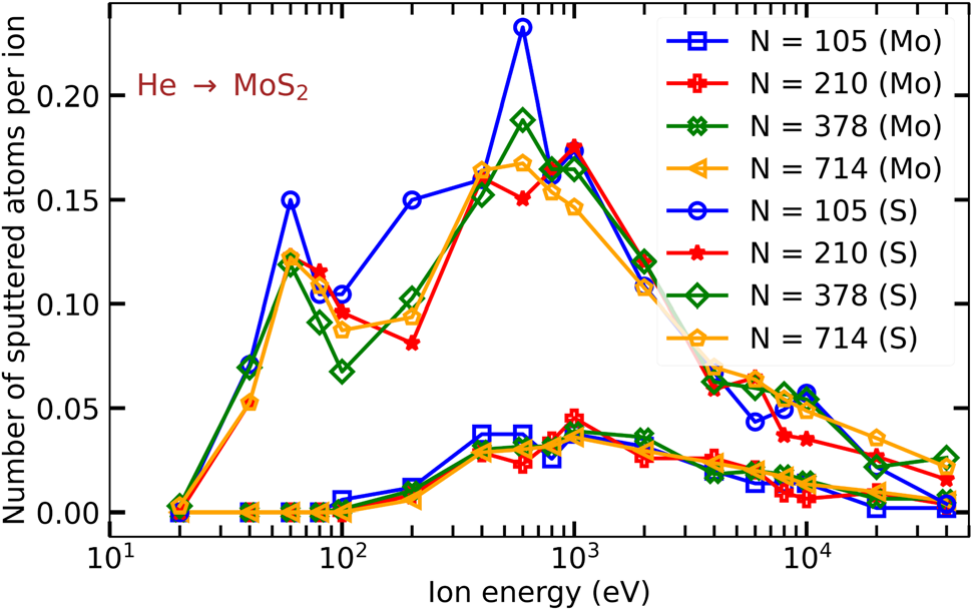
Average number of S and Mo atoms sputtered from a free-standing MoS_2_ sheet upon impacts of He ions as functions of He ion energy calculated using different numbers of impact points *N*.

Fig. 3 shows the numbers of Mo and S atoms sputtered from a free-standing MoS_2_ monolayer per He ion as functions of He ion energy using different numbers of impact points *N* in the meshes. The convergence of the results with regard to the number of points is very slow and non-uniform, as the inclusion of even one point at which sputtering occurs (a rare event, especially for He ions with high energies) can affect the average value. However, the qualitative picture does not change. We note that the convergence for Ar ions is much faster, as the cross section for atom displacement is much larger.

**Fig. 4:**
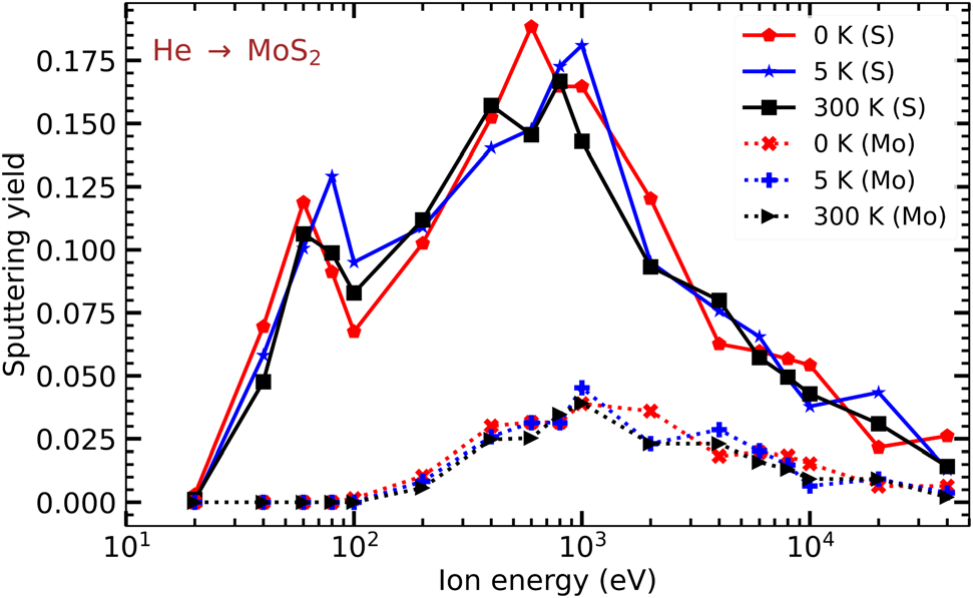
Sputtering yields of Mo and S atoms from a free-standing MoS_2_ monolayer as functions of He ion energy at various temperatures (0, 5, and 300 K).

In Fig. 4, the numbers of Mo and S atoms sputtered from a free-standing MoS_2_ monolayer per He ion are shown as functions of He ion energy at different temperatures: 0 K, 5 K and 300 K. It is evident from the plot that finite temperatures, at least in the considered range, do not have any substantial effect on defect production. Our test calculations for MoS_2_ on a SiO_2_ substrate indicated that at 300 K the results averaged over three different initial velocity distributions are very close to those obtained at zero temperature (about 10% difference), so that finite temperature effects are not expected to change the picture qualitatively or even quantitatively. Taking this into account, we carried out the rest of the simulations at 0 K.

### 3.2 Sputtering yield from different analytical potentials: REBO *vs.* Stillinger–Weber potential

Next, to establish a connection to previous simulations^24,35^ of the response of a free-standing MoS_2_ monolayer to ion irradiation, we calculated the number of sputtered atoms using the REBO and SW potentials and compared the results. The impacts of He and Ar ions were modelled.

**Fig. 5:**
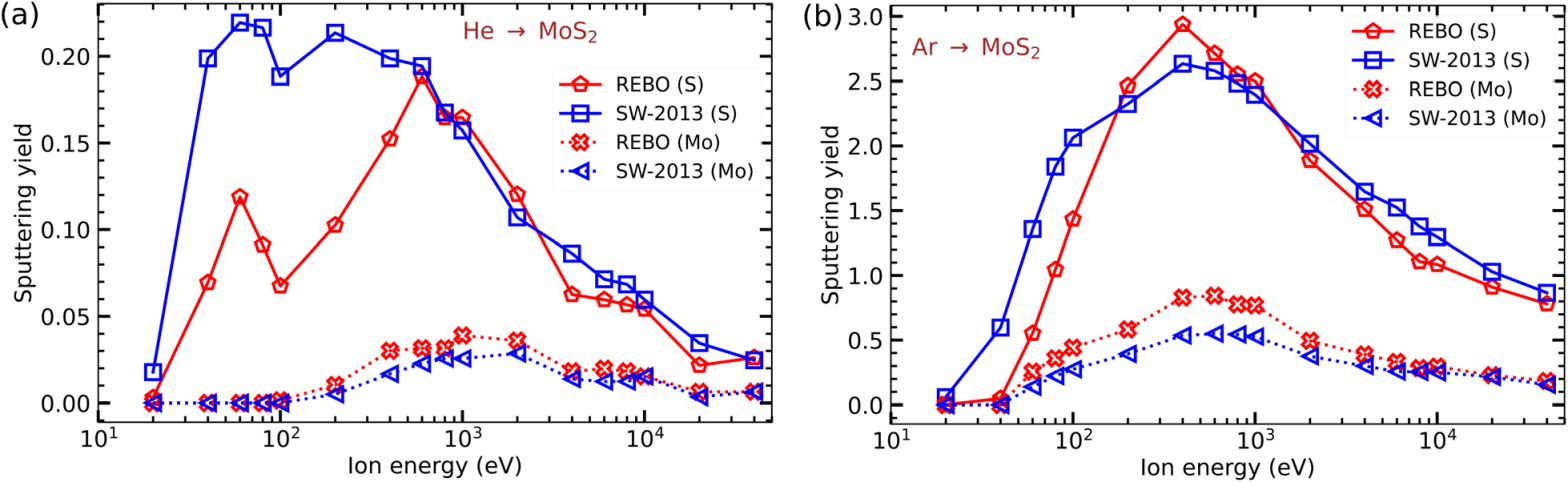
Sputtering yields of Mo and S atoms from a free-standing MoS_2_ monolayer calculated as functions of ion energy for (a) He and (b) Ar ions using the SW and REBO potentials.

For He ions, the calculation results are presented in Fig. 5(a). It is evident that at low He ion energies (below 200 eV) the numbers of sputtered S atoms per ion obtained using the SW potential are noticeably higher than those from the REBO potential. Additionally, the two peaks in the S atom sputtering are less pronounced in the case of SW potential. The results from both potentials are close to each other for He ions at energies above 0.8 keV. Contrary to S sputtering, Mo sputtering is slightly higher for the REBO potential. Such a behavior is related to the differences in the displacement threshold energies of S and Mo atoms. In Table 1, the displacement threshold energies (*T*_d_) calculated using SW and REBO potentials, as well as density functional theory (DFT) are given. The *T*_d_[*S*] values from REBO are higher by almost a factor of two than those from SW. Also, as calculated by Wen *et al.*,^50^ the cohesive energy per unit cell from DFT, SW and REBO potential are 15.90 eV, 12.76 eV and 21.48 eV, respectively. This provides a qualitative explanation for the observed differences in the amount of damage calculated from these potentials. We note that both potentials likely underestimate the number of sputtered Mo atoms.

**Table 1:** Displacement threshold (*T*_d_) values for Mo and S atoms calculated from DFT and classical potentials

	*T*_d_[*S*] (eV)	*T*_d_[Mo] (eV)
DFT	6.9 (ref. 51)	∼ 20 (ref. 51)
SW	5	28
REBO	9.5	41.5

We note that for He ions with energies exceeding 10 keV the number of sputtered atoms is higher from this study than reported previously.^24,35^ This is related to a very small cross section for displacing S atoms at high He ion energies and the insufficient density of the impact points used in previous calculations, as defects are produced only when He ions at such energies collide near head-on with the target atoms, but substantial number of atoms can be sputtered away. The results showed a tendency toward convergence when more than 600 impact points per irreducible area were used. This is irrelevant for Ar ions, which have a much larger cross section for displacing S/Mo atoms.

In Fig. 5(b), a comparison between the REBO and SW potentials for Ar ions is presented. The number of sputtered S atoms is again higher for the SW potential than the REBO potential at Ar ion energies below 100 eV. The calculated amounts of damage obtained from both potentials are comparable for energies above 1 keV. One can conclude that at high (above 1 keV) energies both potentials give similar results. However, because the REBO potential better describes the energetics of defects in MoS_2_, as demonstrated previously (see Fig. 2 in ref. 35), in what follows we use the REBO potential only.

### 3.3 He ion irradiation of monolayer MoS_2_ on SiO_2_ and Au substrates

#### 3.3.1 MoS_2_ on a SiO_2_ substrate

Having analyzed the formation of defects in free-standing MoS_2_ under ion irradiation, we move on to the response of supported material to ion bombardment. We stress that contrary to the previous work^24^ where the atomic structure of the substrate was not accounted for and its influence on the ions and sputtered atoms were modelled by introducing an external repulsive potential, here we explicitly simulate the interaction of the projectiles and 2D material recoil atoms with the substrate atoms. We model ion impacts into a pristine target system and defects at the substrate surface, such as step edges or voids, are not accounted for either. In further discussions, sputtering yield is defined as the ratio of the number of atoms lost from a sheet to the number of incident energetic particles striking the surface. Fig. 2(e) illustrates the criteria used to classify target atoms as either sputtered or remaining in the system after ion irradiation.

**Fig. 6:**
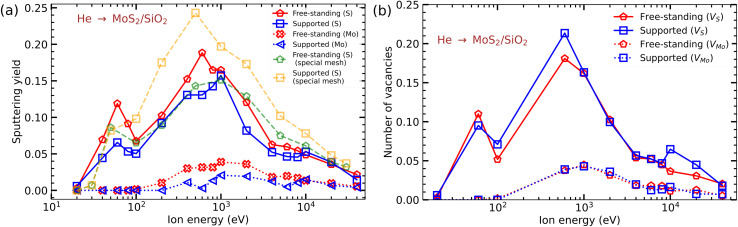
(a) Mo and S atoms sputtering yields from a free-standing MoS_2_ monolayer on a SiO_2_ substrate as functions of He ion energy. (b) The number of Mo and S vacancies produced in the system per ion impact as a function of He ion energy.

In Fig. 6(a) the yields *Y* of Mo and S atoms sputtered from a monolayer MoS_2_ on a SiO_2_ substrate are shown as functions of ion energy. As discussed previously, the presence of an underlying substrate can increase or decrease the defect production in the 2D layer. The calculations were performed using a uniform grid of about 378 impact points per irreducible area for both suspended and supported cases. At high energies, a denser grid of about 700 impacts points was used, and qualitatively similar results were obtained. From the MD simulations using a uniform grid, we found that in the presence of the underlying SiO_2_ substrate, *Y* from a monolayer MoS_2_ is decreased between the ion energy range of 20 eV to 8 keV as compared to the free-standing MoS_2_ and becomes comparable for ion energies of 10 and 20 keV. The decrease in *Y* at low energies is due to the reduction in forward sputtering of S and Mo atoms in the presence of the substrate. This decrease in sputtering yield could have been compensated by the sputtering of Mo and S atoms by recoiled Si and O atoms and back-scattered He ions at energies ≥100 eV, but this is not the case for a uniformly chosen mesh of impact points.

In contrast, when accounting for the contribution of back-scattered ions and using the special mesh, that is sampling the points predominantly close to the atoms in the 2D target and on top of the atoms in the substrate (and rescaling the results according to the sampled area fraction) the data show an opposite trend: the values of *Y* can be about 30% higher due the presence of the substrate, detailed values are provided in Table 2.

**Table 2:** Sulfur sputter yield from a special mesh: contribution of substrate atoms Δ*Y*(SiO_2_) = *Y*(SiO_2_) – *Y*(free); contribution of back-scattered He ions Δ*Y*_BS_ = *p*_BS_·*Y*_BS_; total sulfur sputtering on substrate *Y*(total) = *Y*(SiO_2_) + Δ*Y*_BS_ = *Y*(free) + Δ*Y*(SiO_2_) + Δ*Y*_BS_; total change relative to free-standing material Δ*Y*(total) = Δ*Y*(SiO_2_) + Δ*Y*_BS_; relative changes w.r.t. *Y*(free)

Energy [keV]	*Y*(free)	*Y*(SiO_2_)	Δ*Y*_BS_	*Y*(total)	Δ*Y*(SiO_2_)/*Y*	Δ*Y*_BS_/*Y*	Δ*Y*(total)/*Y*
0.02	0.000	0.000	0.000	0.000	0.0%	0.0%	0.0%
0.03	0.007	0.007	0.000	0.007	0.0%	0.0%	0.0%
0.05	0.086	0.082	0.000	0.082	−4.1%	0.0%	−4.1%
0.1	0.065	0.098	0.000	0.098	+51.3%	0.0%	+51.3%
0.2	0.089	0.175	0.000	0.175	+96.0%	0.0%	+96.0%
0.5	0.143	0.243	0.000	0.243	+69.5%	0.0%	+69.5%
1	0.151	0.181	0.016	0.197	+19.7%	+10.4%	+30.1%
2	0.129	0.152	0.021	0.173	+17.2%	+16.2%	+33.3%
5	0.075	0.088	0.015	0.102	+16.2%	+19.7%	+35.9%
10	0.061	0.070	0.008	0.078	+15.7%	+13.4%	+29.0%
20	0.039	0.045	0.003	0.048	+13.5%	+7.5%	+21.0%
30	0.032	0.037	0.010	0.046	+13.7%	+29.8%	+43.5%

As defects in the 2D target can be created by the sputtered atoms, see Fig. 1, the outcome of the simulations using the second approach should obviously depend on sputtering yield and kinetic energies of the atoms coming from the substrate. In Table 3 we present the sputtering yields of Si and O atoms at 100 eV, 250 eV, 8 keV and 40 keV calculated using the TRIDYN^52^ code and MD simulations. The available experimental data, the MD simulations, and the TRIDYN simulation data, for the substrate alone show fairly good agreement for the amount of sputtered substrate atoms. The abundance of recoils from the substrate indicates that they should contribute to sputtering for the whole ion energy range as confirmed by the increase in sputtering for the special mesh. The reason that for He ions we do not observe these sputtering events caused by substrate atoms on the uniform mesh is that they involve at least three collisions; one with the substrate atom, one reversing its velocity and one with the 2D target on top. Since the interaction cross-section with He ions is small we need to sample within the close vicinity of the atoms. In fact we find that atom-centered sampling shows an increase in sputter yield for the supported material (even on a per-impact point comparison). Table 3 further suggests that back-scattered He ions should contribute at higher ion energies. He ions with energies below 100 eV will not cause significant damage in MoS_2_, meaning backscattering becomes relevant for primary ion energies above 5 keV. Since even for the increased special mesh sampling we do not observe He ions being backscattered, for this energy range we explicitly account for back-scattering by starting the ion from underneath the substrate region with the velocity vector pointing towards the surface and kinetic energy as computed from TRIDYN simulations (impact points are uniformly selected from a 10 Å × 10 Å rectangular region). In this way it travels through the substrate potentially causing a small cascade of substrate atoms being sputtered or interacting with the 2D target directly. The resulting contribution to the sputter yield, scaled by the backscattering probability, is comparable to the effect of the sputtered substrate atoms. Altogether, our results indicate that although the presence of the substrate does not qualitatively change the dependence of sputtering yield on He ion energy we find an overall increase in sputtering yield for the supported 2D material, when using the special mesh and accounting for back-scattering.

**Table 3:** Substrate sputtering yields *Y*_sub_ and back-scattered ions *Y*_BS_ from bare Au and SiO_2_ under normal incidence of He ions, as obtained from TRIDYN simulations and MD simulations. For TRIDYN the average energy upon leaving the substrate is given for sputtered recoils 〈*E*_sub_〉 and back-scattered ions 〈*E*_BS_〉. Available experimental data are also included for comparison

Target	Energy [keV]	Exp. *Y*_sub_	MD *Y*_sub_	TRIDYN		MD *Y*_BS_	TRIDYN	
				*Y*_sub_	〈*E*_sub_〉 [eV]		*Y*_BS_	〈*E*_BS_〉 [eV]
SiO_2_	0.1		0.05	0.05	5.9	0.348	0.205	30
	0.25	0.050 (ref. 55)	0.082	0.085	11.2		0.170	80
	8	0.079 (ref. 55)	0.042	0.051	45.1	0.005	0.032	1800
	40		0.024	0.019	55.1	0.000	0.004	6300
Au	0.1		0.038	0.004	1.3	0.645	0.604	60
	0.2	0.02 (ref. 56)	0.183	0.027	3.2		0.571	120
	8		0.096	0.131	19.7	0.019	0.344	3700
	40		0.021	0.079	28.1	0.010	0.169	14 000
	45	0.046 (ref. 57)	0.015	0.074	25.7		0.157	15 400

As not all sputtered atoms leave the system, but can be adsorbed on the MoS_2_ monolayer or form other defects,^29^ the number of produced vacancies per ion impact can be higher than the sputtering yield. In Fig. 6(b) the numbers of single S (*V*_S_) and single Mo (*V*_Mo_) vacancies produced per ion impact are shown. The analysis of the atomic configurations after ion irradiation indicated that, in addition to single, there are double S vacancies, but their concentration is an order of magnitude lower. Furthermore, as reported in previous studies, the migration barriers for S and Mo diffusion on the MoS_2_ monolayer are 1.67 eV^53^ and 0.62 eV,^54^ respectively. These barriers are high enough for inhibiting adatom diffusion (especially S adatoms) at room temperature. At the same time, our calculations using the REBO potential give very low adsorption energies as compared to the DFT results (0.08 *vs.* 2.2 eV) for S atoms, indicating that diffusivity of adatoms in this model can be high through the desorption/adsorption mechanism. We note, however, that the DFT migration barriers were obtained for a free-standing MoS_2_ sheet. It has also been reported^53^ that the barriers for the migration of interstitials in the multi-layer structure strongly decrease, as the interaction of the migrating species with the environment lowers the energies of the configurations. The substrate may also have a similar effect, but due to a multitude of different configurations (especially for amorphous substrates) and the extremely long simulation times required to model the evolution of the system at a macroscopic time scale, detailed simulations are beyond the scope of this study.

#### 3.3.2 MoS_2_ on a Au substrate

**Fig. 7:**
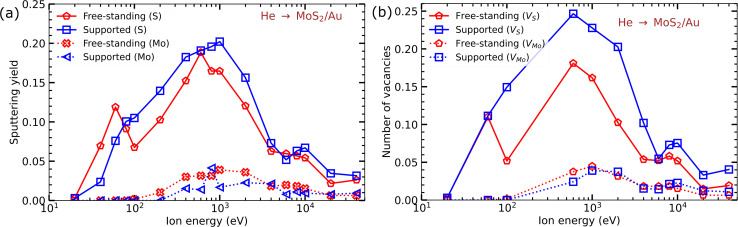
(a) Sputtering yields of Mo and S atoms from free-standing and MoS_2_ on the Au (111) substrate per as functions of He ion energy. (b) The number of Mo and S vacancies produced in the system per ion impact as a function of He ion energy.

As MoS_2_ sheets can also be grown and irradiated on metal^11,58^ substrates including gold,^59^ we also studied the response of the MoS_2_ sheet on the (111) Au surface to ion bombardment. Fig. 7(a) shows the sputtering yields *Y* for Mo and S atoms sputtered from a monolayer MoS_2_ on the Au substrate as functions of ion energy. The overall trends are similar to those observed for MoS_2_ on a SiO_2_ substrate; for most He ion energies more defects are produced for the supported system than for the free-standing one. Table 3 presents the available experimental data on sputtering yield, along with TRIDYN and MD results. As compared to the SiO_2_ surface, the yield is not much different, but the analysis of He ion trajectories and energies of sputtered Au atoms indicates that the back-scattered He ions are the main source of the enhanced damage in the supported system, as most of the sputtered Au atoms do not have enough energy to displace S atoms from the MoS_2_ monolayer.

### 3.4 Ar ion irradiation of the MoS_2_ monolayer on SiO_2_ and Au substrates

**Fig. 8:**
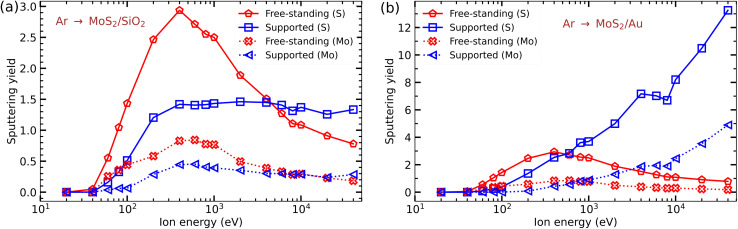
Sputtering yields of Mo and S atoms as functions of Ar ion energies for suspended MoS_2_ and MoS_2_ on the SiO_2_ substrate (a) and Au substrate (b).

We also studied the response of MoS_2_ sheets to heavy ion irradiation and chose Ar ions for this purpose. A uniform mesh of impact points (210–378 points per irreducible area) was used. Fig. 8(a) and (b) show the sputtering yield from the MoS_2_ monolayer on SiO_2_ and Au substrates. It is evident that the amount of damage in MoS_2_ caused by Ar ion irradiation is quite different for the free-standing and supported systems. For both SiO_2_ and Au substrates, the sputtering yield of S and Mo atoms is higher in the free-standing system at low ion energies, and the opposite is true at energies above 10 keV.

#### 3.4.1 MoS_2_ on the SiO_2_ substrate

Specifically, for MoS_2_ sheets on the SiO_2_ substrate the number of sputtered S atoms is reduced as compared to the free-standing case until the energy reaches 4 keV. This decrease is due to a reduction in forward sputtering of Mo and S atoms by the substrate, as schematically depicted in Fig. 1(a). For suspended MoS_2_, the sputtering yields of S and Mo atoms start decreasing as ion energy enters the keV range (1–40 keV), while for the MoS_2_/SiO_2_ case, the S sputtering yield increases rapidly until 400 eV and becomes nearly constant at higher energies (up to 40 keV). From Table 4, the sputtering yields of Si and O atoms by low energy Ar ions (100 eV) from MD simulations is calculated to be 0.08. The results are in very good agreement with the experimental values. Similarly, for high energy Ar ions, the total number of Si and O atoms sputtered per ion is 1.13 (40 keV) from simulations and 1.6 (32 keV) in the experiment. Taking into account a high sputtering yield from the substrate, one can conclude that the production of defects in MoS_2_ sheets on the SiO_2_ substrate is governed by the substrate due to the collisions of the sputtered substrate atoms. At low ion energies back-scattered Ar ions also contribute to defect production, see Table 4.

**Table 4:** Substrate sputtering yields *Y*_sub_ and back-scattered ions *Y*_BS_ from bare Au and SiO_2_ under normal incidence of Ar ions, as obtained from TRIDYN simulations and MD simulations. For TRIDYN the average energy upon leaving the substrate is given for sputtered recoils 〈*E*_sub_〉 and back-scattered ions 〈*E*_BS_〉. Available experimental data are also included for comparison

Target	Energy	Exp.	MD	TRIDYN		MD	TRIDYN	
	[keV]	*Y*_sub_	*Y*_sub_	*Y*_sub_	〈*E*_sub_〉 [eV]	*Y*_*BS*_	*Y*_*BS*_	〈*E*_BS_〉 [eV]
SiO_2_	0.1	0.08 (ref. 60)	0.085	0.027	4.6	0.415	0.00003	6.7
	1			0.602	15.7		0.00020	17.7
	10			1.284	51.5		0.00018	110
	40	(32 keV) 1.6 (ref. 61)	1.130	1.251	108.5	0.000	0.00004	270
Au	0.1	0.32 (ref. 62)	0.448	0.275	6.5	1.000	0.417	33.9
	1			2.138	16.5		0.292	280
	10			4.608	50.1		0.212	2700
	40	(45 keV) 5.8 (ref. 57)	6.982	4.888	69.1	0.055	0.159	10 600

#### 3.4.2 MoS_2_ on the Au substrate

Similar to the SiO_2_ substrate, the sputtering yield of S and Mo atoms from the MoS_2_/Au system is lower than that for the free-standing material at low Ar ion energies. However, the substrate starts playing a more important role at lower energies, about 700 eV. The sputtering of both S and Mo atoms in the supported system increases dramatically for Ar ion energies above 1 keV as shown in Fig. 8(b). The MD simulations of the response of the substrate to ion irradiation indicate that sputtering of the substrate atoms is the main reason for the enhancement in defect production, see Table 4; the sputtering yield of Au by 40 keV Ar ions is 6.98 Au atoms per ion compared to 0.45 Au atoms per ion by 100 eV Ar ions. Hence, we can conclude that for Ar irradiation, the Au substrate has a dramatic influence on the defect production in the adsorbed monolayer.

### 3.5 He and Ar irradiation of graphene on SiO_2_ and Au substrates

**Fig. 9:**
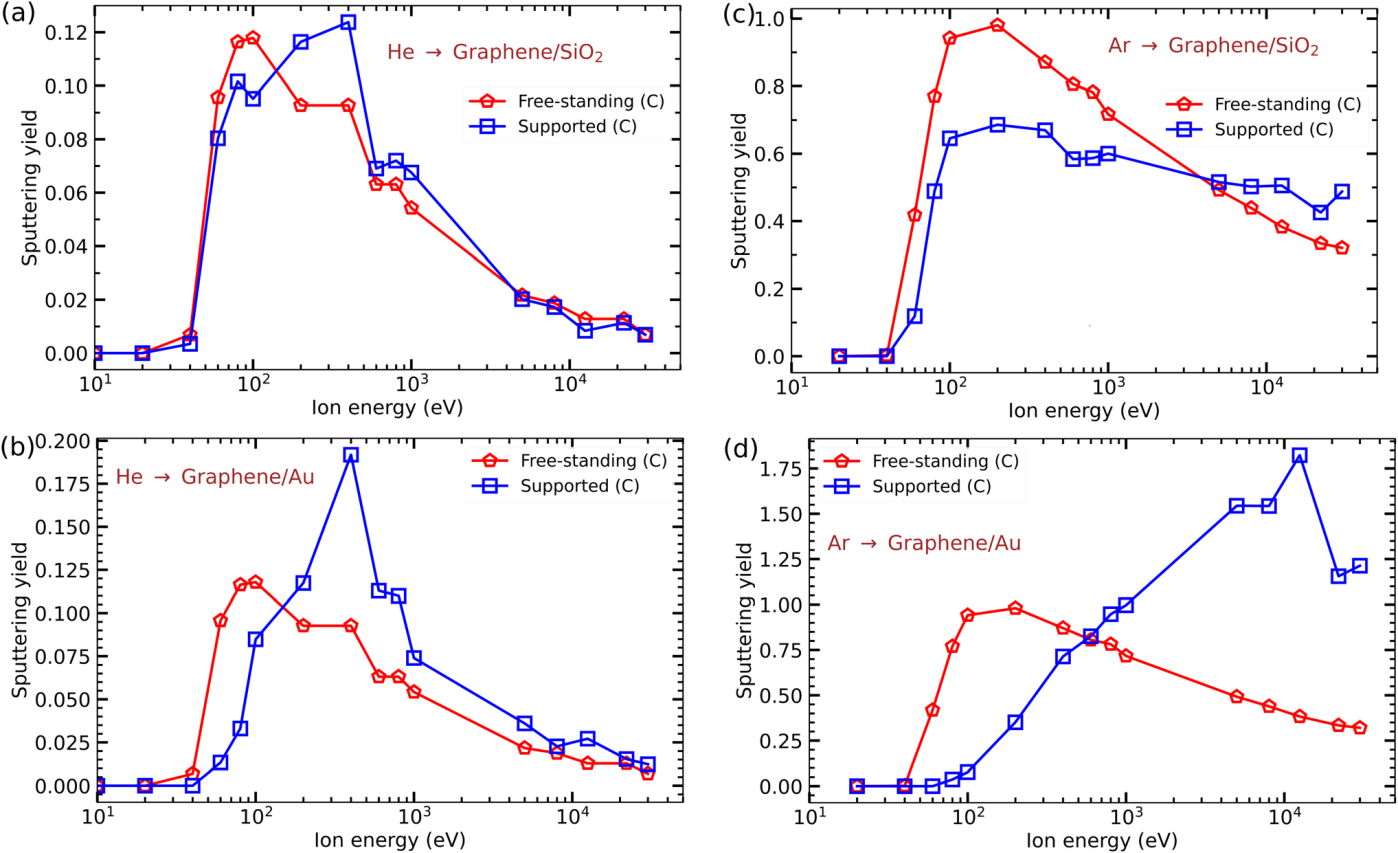
Sputtering yields of C atoms from suspended and supported graphene as functions of He and Ar ion energies. (a) Impacts of He ions onto graphene on the SiO_2_ substrate. (b) Impacts of He ions onto graphene on the Au substrate. (c) Impacts of Ar ions onto graphene on the SiO_2_ substrate. (d) Impacts of Ar ions onto graphene on the Au substrate.

Finally we studied the irradiation response of graphene on SiO_2_ and Au substrates, explicitly accounting for their atomic structures and compared the results to those obtained previously^24^ by a combination of MD and binary collision Monte Carlo methods, where the effects of substrate were included by introducing an external repulsive potential acting on the ion and displaced atoms. As compared to the previous study, a much denser grid (378 impact points per irreducible area) was used. The results of the calculations are shown in Fig. 9.

Fig. 9(a) presents the sputtering yield of C atoms from graphene on the SiO_2_ substrate under He ion irradiation, and Fig. 9(b) shows the results for the Au substrate. It is evident that, similar to MoS_2_, the substrate decreases defect production at low energies (below 100 eV), although the effect is not so strong in the case of the SiO_2_ substrate. In contrast, the substrates increase the production of defects in the energy range of 200 eV to 1 keV, with a maximum at about 400 eV. At higher ion energies (≥5 keV), the difference between free-standing and supported sputtering yields is rather small. We note that at high He ion energies (above 10 keV) sputtering yield from free-standing graphene calculated with a much higher number of impact points is larger than reported previously.^24^

We also carried out similar simulations for graphene on SiO_2_ and Au substrates irradiated with Ar ions. The results are shown in Fig. 9(c and d). We found that both substrates significantly influence the production of defects, especially the Au substrate. At low ion energies less defects are created in the supported graphene, but at high energies (≥5 keV) sputtering yield is lower in the free-standing system, especially for the Au substrate.

## 4 Conclusions

To conclude, using analytical potential MD simulations we studied the production of defects in single layer graphene and MoS_2_, two archetypal 2D materials, both free-standing and supported, under He and Ar ion irradiation across a wide range of energies. We explicitly took into account the atomic structure of the substrate and used several approaches to choose impact points in the supercell. Our results indicate that while for heavy ions like Ar a relatively small number of impact points is sufficient to adequately describe damage production, a much larger number of impact points is required for He ion with ion energies exceeding 10 keV, typical of helium ion microscopy. We further suggested a special approach for selecting the impact points, which is based on the selection of the impact points corresponding to the ion trajectories with a high likelihood for defect production followed by the rescaling of the results as the ratio of the impact point area to the total area of the target. We also investigated the effect of finite (room) temperature on the formation of defects under irradiation, and found it to be negligible. Finite temperatures should affect the *in situ* annealing of defects, especially in supported 2D materials, when displaced atoms can diffuse between the 2D material and the substrate. *In situ* annealing will reduce the number of defects, but as it happens on a macroscopic time scale, which cannot be accounted for in the MD simulations, our data should be considered as the upper limit on the concentration of irradiation-induced defects.

Our results indicate that for free-standing 2D materials there is a maximum on the “number of defects *vs.* ion energy” curve for both He and Ar ions, in line with the previous findings.^23,24,35^ This is also true for the supported materials under He ion irradiation, but the values for high ion energies are higher than those previously reported, as better statistics, that is a larger number of impact points, were used. In the case of Ar ions, the substrate can decrease defect production at low energies or enhance it at higher energies for the considered 2D materials on both SiO_2_ and Au substrates. The effect is particularly strong for the Au substrate, which in contrast to the SiO_2_ substrate,^23^ has never been modelled before. Overall, our simulations provide microscopic insights into different channels of defect production in free-standing and supported 2D systems, and yield quantitative results which can be directly compared to experimental data.

## Conflicts of interest

There are no conflicts to declare.

## Data Availability

The code LAMMPS used in the simulations can be found at https://www.lammps.org, see also https://doi.org/10.1016/j.cpc.2021.108171. The version of the code employed for this study is version 8/24.
